# Supplementary material for: Exploring the effects of Dasatinib, Quercetin, and Fisetin on DNA methylation clocks: a longitudinal study on senolytic interventions
Source: Aging (Albany NY). 2024 Feb 22;16(4):3088–106. doi: 10.18632/aging.205581 (PMC10929829; doi:10.18632/aging.205581)
Supplement: Supplementary Figure 1 [file aging-16-205581-s001.pdf]

SUPPLEMENTARY FIGURE

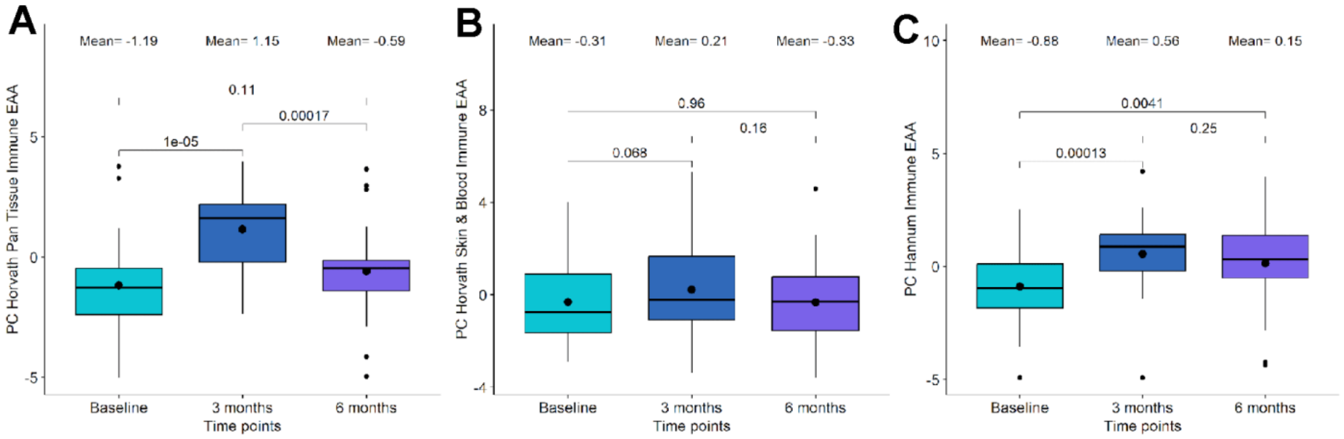

Supplementary Figure 1. Boxplot showing the evolution of epigenetic age acceleration (EAA) first-generation clocks after adjusting by immune cells in the Dasatinib and Quercetin (DQ) study. (**A**) PC Horvath pan tissue Immune EAA. (**B**) PC Horvath Skin and Blood Immune EAA. (**C**) PC Hannum Immune EAA. In the X-axis, the time points of measurements, in the Y axis, the EAA measure adjusted by immune cells. On the top, the mean values at each time point and the p-values of the paired t-tests. The box represents the interquartile range (IQR) with the median represented as a horizontal line and the mean as the dot. The vertical lines show the minimum and maximum values. When outliers are identified, those values are represented as dots.
