# Supplementary material for: Exploring the effects of Dasatinib, Quercetin, and Fisetin on DNA methylation clocks: a longitudinal study on senolytic interventions
Source: Aging (Albany NY). 2024 Feb 22;16(4):3088–106. doi: 10.18632/aging.205581 (PMC10929829; doi:10.18632/aging.205581)
Supplement: Supplementary Table 1 [file aging-16-205581-s002.pdf]

SUPPLEMENTARY TABLES

Supplementary Table 1. Differentially methylated loci for Dasatinib and Quercetin (DQ) treatment and Dasatinib, Quercetin, and Fisetin (DQF) treatment.

**Dasatinib and Quercetin**					
**0-3 months**					
**CpG site**	**logFC**	**diffMeth**	**P-Value**	**FDR**	**geneID**
cg05915794	0.2878	Hyper	$1.5 \cdot 10^{-12}$	$1.3 \cdot 10^{-6}$	HRNBP3
cg18889307	-0.08974	Hypo	$6.9 \cdot 10^{-9}$	0.002	TGIF1
cg02889828	-0.1833	Hypo	$8.6 \cdot 10^{-9}$	0.002	
cg27262015	-0.1243	Hypo	$2.0 \cdot 10^{-8}$	0.004	SORBS2
cg04704414	0.1107	Hyper	$1.3 \cdot 10^{-7}$	0.022	NUBP2
cg06902281	0.1175	Hyper	$1.9 \cdot 10^{-7}$	0.027	ZNF169
cg04955246	-0.155	Hypo	$2.5 \cdot 10^{-7}$	0.031	PRKCA
cg02840515	-0.1262	Hypo	$4.3 \cdot 10^{-7}$	0.039	KIAA2012
cg04132292	-0.2873	Hypo	$4.4 \cdot 10^{-7}$	0.039	
cg25024143	-0.1463	Hypo	$4.6 \cdot 10^{-7}$	0.039	SP2
cg20273352	-0.1645	Hypo	$5.6 \cdot 10^{-7}$	0.044	IGHMBP2
**0-6 months**					
**CpG site**	**logFC**	**diffMeth**	**P-Value**	**FDR**	**geneID**
cg10585661	-0.1102	Hypo	$8.0 \cdot 10^{-8}$	0.035	FAM131A
cg05779406	0.271	Hyper	$8.1 \cdot 10^{-8}$	0.035	ZFAND2A
**Dasatinib, Quercetin, and Fisetin**					
**0-6 months**					
**CpG site**	**logFC**	**diffMeth**	**P-Value**	**FDR**	**geneID**
cg00379708	-0.1492	Hypo	$1.4 \cdot 10^{-9}$	$6.7 \cdot 10^{-4}$	RBM20
cg13172627	0.5832	Hyper	$1.9 \cdot 10^{-9}$	$6.7 \cdot 10^{-4}$	VENTXP1
cg26676360	0.1803	Hyper	$2.3 \cdot 10^{-9}$	$6.7 \cdot 10^{-4}$	LRP1
cg02632099	0.3497	Hyper	$3.5 \cdot 10^{-9}$	$7.5 \cdot 10^{-4}$	
cg18054725	-0.1509	Hypo	$9.2 \cdot 10^{-9}$	0.002	ICAM3
cg01613010	0.2131	Hyper	$2.5 \cdot 10^{-8}$	0.002	
cg16640929	-0.6613	Hypo	$2.6 \cdot 10^{-8}$	0.002	ZBTB12
cg00877151	0.4233	Hyper	$2.8 \cdot 10^{-8}$	0.002	IPO11-LRRC70;IPO11
cg06310713	0.3591	Hyper	$3.1 \cdot 10^{-8}$	0.002	PALLD
cg08187458	0.1809	Hyper	$3.3 \cdot 10^{-8}$	0.002	
cg00288652	-0.1792	Hypo	$3.5 \cdot 10^{-8}$	0.002	
cg04706867	-0.1987	Hypo	$3.6 \cdot 10^{-8}$	0.002	CNTNAP2
cg09093137	-0.5388	Hypo	$3.7 \cdot 10^{-8}$	0.002	SRPK2
cg03012879	-0.4304	Hypo	$3.7 \cdot 10^{-8}$	0.002	HMGCR
cg12993163	-0.1746	Hypo	$4.2 \cdot 10^{-8}$	0.002	SHOX2

All the significant probes (FDR<0.05) for DQ analyses are included. However, only the top 15 probes are included in the DQF analysis.
