# Supplementary material for: Exploring the effects of Dasatinib, Quercetin, and Fisetin on DNA methylation clocks: a longitudinal study on senolytic interventions
Source: Aging (Albany NY). 2024 Feb 22;16(4):3088–106. doi: 10.18632/aging.205581 (PMC10929829; doi:10.18632/aging.205581)
Supplement: Supplementary Table 3 [file aging-16-205581-s004.docx]

**Supplementary Table 3. Statistical analysis for comparing baseline and 6-month methylation risk score surrogates in the Dasatinib, Quercetin, and Fisetin study.**

|  | **Mean** | | **Baseline vs 6-month** | |
| --- | --- | --- | --- | --- |
|  | **Baseline** | **6-month** | **T-score** | **P-value** |
| **Epigenetic Age Zhang.** | -0.679 | -0.556 | -0.719 | 0.492 |
| **Alcohol** | -11.613 | -11.406 | -2.928 | 0.019 |
| **Body Fat** | -8.756 | -8.987 | 0.247 | 0.811 |
| **Body Mass Index** | -0.571 | -0.58 | 0.36 | 0.728 |
| **HDL Cholesterol** | 2.598 | 2.612 | -0.313 | 0.762 |
| **Smoking** | 2.699 | 2.581 | 1.45 | 0.185 |
| **Waist.Hip.Ratio** | -0.323 | -0.306 | -1.891 | 0.095 |
| **ADAMTS** | 0.1 | 0.104 | -1.311 | 0.226 |
| **Adiponectin** | -0.072 | -0.073 | 0.49 | 0.637 |
| **Afamin** | -0.01 | -0.01 | -0.279 | 0.787 |
| **Alpha L iduronidase** | 0.112 | 0.112 | -0.082 | 0.937 |
| **Aminoacylase 1** | -0.319 | -0.322 | 0.687 | 0.511 |
| **B2 microglobulin** | -0.376 | -0.376 | 0.066 | 0.949 |
| **BMP.1** | 0.115 | 0.121 | -2.644 | 0.03 |
| **CCL11** | -0.003 | -0.004 | 0.362 | 0.726 |
| **CCL17** | -0.428 | -0.431 | 1.266 | 0.241 |
| **CCL18** | -0.145 | -0.144 | -0.892 | 0.399 |
| **CCL21** | -0.131 | -0.133 | 0.968 | 0.362 |
| **CCL22** | -0.065 | -0.066 | 0.337 | 0.745 |
| **CCL25.C.C** | -0.061 | -0.062 | 1.089 | 0.308 |
| **CD163** | -0.246 | -0.244 | -0.666 | 0.524 |
| **CD209.antigen** | 0.134 | 0.139 | -1.453 | 0.184 |
| **CD48.antigen** | -0.157 | -0.158 | 0.267 | 0.797 |
| **CD6** | 0.097 | 0.096 | 0.342 | 0.741 |
| **CDL5** | -0.069 | -0.07 | 0.234 | 0.821 |
| **CHIT.1** | -0.173 | -0.173 | -0.071 | 0.945 |
| **CLEC11A.e1** | -0.005 | -0.015 | 1.792 | 0.111 |
| **CLEC11A.e2** | -0.145 | -0.153 | 1.798 | 0.11 |
| **Coagulation.factor.VII** | 0.049 | 0.048 | 0.353 | 0.733 |
| **Complement.C4** | 0.036 | 0.037 | -1.052 | 0.324 |
| **Complement.C5a** | 0.159 | 0.161 | -0.498 | 0.632 |
| **Complement.c9** | -0.009 | -0.013 | 0.756 | 0.471 |
| **Contactin.4** | 0.171 | 0.17 | 0.386 | 0.71 |
| **CRP** | -0.113 | -0.117 | 1.396 | 0.2 |
| **CRTAM** | 0.053 | 0.055 | -0.797 | 0.448 |
| **CXCL10** | 0.137 | 0.141 | -0.95 | 0.37 |
| **CXCL10.soma** | -0.347 | -0.346 | -0.206 | 0.842 |
| **CXCL11** | 0.097 | 0.101 | -0.703 | 0.502 |
| **CXCL11.soma** | -0.056 | -0.054 | -0.788 | 0.453 |
| **CXCL9** | -0.037 | -0.035 | -1.532 | 0.164 |
| **E.selectin** | -0.026 | -0.026 | -0.018 | 0.986 |
| **Ectodysplasin.A** | -0.216 | -0.219 | 1.531 | 0.164 |
| **EN.RAGE** | 0.051 | 0.047 | 1.278 | 0.237 |
| **ENPP7** | -0.017 | -0.015 | -0.203 | 0.844 |
| **ESM.1** | -0.244 | -0.247 | 1.814 | 0.107 |
| **EZR** | -0.019 | -0.018 | -0.495 | 0.634 |
| **FAP** | -0.143 | -0.141 | -1.418 | 0.194 |
| **FCER2** | -0.251 | -0.252 | 0.271 | 0.793 |
| **FCGR3A** | -0.203 | -0.207 | 1.06 | 0.32 |
| **FcRL2** | -0.175 | -0.176 | 0.479 | 0.645 |
| **FGF.21** | -0.116 | -0.113 | -1.642 | 0.139 |
| **G.CSF** | -0.015 | -0.016 | 0.418 | 0.687 |
| **Galectin.4** | -0.208 | -0.208 | -0.302 | 0.771 |
| **GDF.8** | 0.141 | 0.142 | -0.852 | 0.419 |
| **GHR** | 0.139 | 0.141 | -0.643 | 0.538 |
| **GPIba** | -0.362 | -0.363 | 0.141 | 0.892 |
| **Granulysin** | -0.115 | -0.112 | -0.76 | 0.469 |
| **Granzyme.A** | -0.034 | -0.035 | 0.326 | 0.753 |
| **GZMA** | -0.067 | -0.068 | 0.762 | 0.468 |
| **HCII** | 0.039 | 0.038 | 0.084 | 0.935 |
| **HGF** | 0.043 | 0.041 | 0.824 | 0.434 |
| **HGFA** | 0.358 | 0.359 | -0.499 | 0.631 |
| **HGFI** | 0.551 | 0.56 | -0.88 | 0.405 |
| **ICAM5** | -0.092 | -0.093 | 0.203 | 0.844 |
| **IGFBP.1** | -0.124 | -0.125 | 0.283 | 0.784 |
| **IGFBP.4** | -0.048 | -0.045 | -3.234 | 0.012 |
| **Insulin.receptor** | -0.136 | -0.137 | 0.54 | 0.604 |
| **Interleukin.19** | -0.011 | -0.009 | -0.67 | 0.522 |
| **L.selectin** | 0.051 | 0.051 | -0.206 | 0.842 |
| **LFT** | -0.005 | -0.007 | 0.747 | 0.476 |
| **LGALS3BP** | 0.008 | 0.006 | 1.019 | 0.338 |
| **LY9** | -0.124 | -0.123 | -0.265 | 0.798 |
| **Lymphotoxin.abeta** | -0.037 | -0.034 | -0.991 | 0.351 |
| **MIA** | 0.102 | 0.103 | -0.456 | 0.66 |
| **MMP.1.1** | -0.123 | -0.122 | -0.348 | 0.737 |
| **MMP.12** | -0.201 | -0.201 | 0.01 | 0.992 |
| **MMP.9** | -0.211 | -0.212 | 0.323 | 0.755 |
| **MMP.1** | -0.097 | -0.098 | 0.689 | 0.51 |
| **MRC2** | 0.043 | 0.043 | 0.011 | 0.992 |
| **Myeloperoxidase** | -0.001 | -0.001 | -0.047 | 0.964 |
| **N.CDase** | 0.105 | 0.106 | -0.572 | 0.583 |
| **NCAM.120** | 0.043 | 0.043 | 0.294 | 0.776 |
| **NEP** | -0.037 | -0.031 | -2.053 | 0.074 |
| **NMNAT1** | -0.05 | -0.05 | -0.392 | 0.705 |
| **NOTCH1** | 0.069 | 0.07 | -0.492 | 0.636 |
| **NRTK3** | 0.144 | 0.147 | -1.144 | 0.286 |
| **NTRK3** | 0.136 | 0.135 | 0.234 | 0.821 |
| **OSM** | 0.1 | 0.094 | 1.746 | 0.119 |
| **Osteomodulin** | 0.329 | 0.326 | 1.924 | 0.091 |
| **PAPP.A** | -0.402 | -0.399 | -0.426 | 0.681 |
| **PIGR** | -0.216 | -0.218 | 0.636 | 0.542 |
| **RARRES2** | 0.01 | 0.013 | -1.15 | 0.283 |
| **Resistin** | -0.122 | -0.126 | 0.93 | 0.379 |
| **S100.A9** | 0.035 | 0.034 | 0.345 | 0.739 |
| **Semaphorin.3E** | -0.057 | -0.057 | 0.245 | 0.813 |
| **SERPIN.A3** | 0.157 | 0.157 | 0.156 | 0.88 |
| **SHBG** | -0.068 | -0.07 | 0.834 | 0.428 |
| **SIGLEC1** | -0.043 | -0.042 | -0.524 | 0.614 |
| **SKR3** | 0.163 | 0.164 | -0.485 | 0.641 |
| **SLITRK5** | 0.148 | 0.154 | -1.471 | 0.18 |
| **SMPD1** | -0.034 | -0.034 | 0.06 | 0.953 |
| **Stanniocalcin.1** | -0.04 | -0.037 | -1.222 | 0.257 |
| **Testican.2** | -0.212 | -0.214 | 0.795 | 0.449 |
| **TGF.alpha** | 0.024 | 0.019 | 1.798 | 0.11 |
| **THBS2** | -0.116 | -0.119 | 0.996 | 0.348 |
| **TNFRSF17** | 0.013 | 0.012 | 1.069 | 0.316 |
| **TNFRSF1B** | -0.106 | -0.108 | 1.039 | 0.329 |
| **TPO** | -0.308 | -0.31 | 0.7 | 0.504 |
| **Trypsin.2** | -0.104 | -0.103 | -1.352 | 0.213 |
| **Tryptase.beta.2** | -0.174 | -0.174 | 0.043 | 0.967 |
| **VCAM1** | -0.008 | -0.008 | 0.012 | 0.991 |
| **VEGFA** | 0.125 | 0.124 | 0.255 | 0.805 |

We used methylation risk scores surrogates to predict and quantify predicted changes in circulating proteomic markers, as described in Marioni et al. The first two columns show the mean values for each immune cell proportion at each time point. The next columns have information about the t-test between baseline and 6-month test.
